# The incidence of post operative venous thromboembolism in patients undergoing varicose vein surgery recorded in Hospital Episode Statistics

**DOI:** 10.1308/003588412X13171221592096

**Published:** 2012-10

**Authors:** PA Sutton, Y El-Duhwaib, J Dyer, AJ Guy

**Affiliations:** Mid Cheshire Hospitals NHS Foundation Trust

**Keywords:** Varicose veins, Surgery, Deep vein thrombosis, Pulmonary embolism, Hospital Episode Statistics

## Abstract

**INTRODUCTION:**

The aim of this study was to establish the incidence of post-operative venous thromboembolism (VTE) following varicose vein treatment.

**METHODS:**

Hospital Episode Statistics (HES) data were obtained for all patients undergoing varicose vein treatment between April 2006 and April 2007 to identify those reattending with either deep vein thrombosis or pulmonary embolism within 12 months.

**RESULTS:**

The incidence of VTE was 0.51%, which was comparable with the incidence for those undergoing open surgery (0.54%), sclerotherapy (0.19%) and endovenous laser therapy (EVLT) (0.47%). The incidence of VTE in those undergoing combined EVLT and phlebectomy was 1.26% (*p*=0.01). In contrast to unilateral treatment (all modalities), where bilateral treatment was performed an increase in the incidence of VTE was seen in those undergoing redo (1.62%) and short saphenous system (1.16%) treatments.Overall, 1.02% of cases were performed under local anaesthesia with zero incidence of VTE in this cohort.

**CONCLUSIONS:**

The overall incidence of VTE recorded in HES was 0.51% and appears to be highest in those undergoing bilateral redo or short saphenous system surgery as well as those undergoing a combination of EVLT and phlebectomy. The use of VTE prophylaxis, particularly in these groups, is recommended.

Varicose veins affect approximately 25% of the population with symptoms varying from mild ache through to significant morbidity as a result of ulceration.^1^ Open surgery has been the main treatment for varicose veins for many years but recently there has been an increase in the popularity of a number of ambulatory procedures including but not limited to endovenous laser therapy (EVLT), radiofrequency ablation (RFA) and sclerotherapy. Many studies have shown outcomes comparable with those for traditional surgery, both in terms of patient satisfaction and rates of recurrence.^2^

Patients undergoing surgery are at increased risk of venous thromboembolism (VTE)^3^ and, with varicose veins potentially also being a risk factor, post-operative VTE in this cohort represents a significant clinical problem. There have been a number of small single centre studies performed to attempt to quantify this risk^4–6^ but no large multicentre study or review has been undertaken. Our study aimed to use Hospital Episode Statistics (HES) data to accurately quantify the risk of post-operative VTE in patients undergoing intervention for varicose veins.

## Methods

HES data were obtained for all patients undergoing intervention for varicose veins in NHS trusts throughout England between April 2006 and April 2007. This dataset was employed as it was the most recently available complete dataset that permitted a minimum follow-up period of 12 months. The cohort was followed to identify those patients reattending with either deep vein thrombosis (DVT) or pulmonary embolism (PE) within 12 months of their intervention. Any patient who had been coded for a PE was included in the study as such. If they had been coded for a DVT in addition to this, this information was not used for the analysis.

Data were extracted into an Access® database (Microsoft, Redmond, WA, US) and analysed. The breakdown of the coding for this can be seen in Table 1. Due to the small numbers involved and multiple codes attached to individual patients, EVLT and RFA were combined and analysed as one group. A number of unspecified open surgery codes exist within HES, which we grouped together and referred to as phlebectomy for the purposes of analysis. Statistical analysis was performed using Excel® (Microsoft) with an analysis of proportions and a chi-squared test.

**Table 1:** Hospital Episodes Statistics data codes relevant to treatments for varicose veins

Intervention	Codes
Local anaesthetic procedure	Y82.1, Y82.2, Y82.3, Y83.9
Primary open surgery, long saphenous	L84.1, L85.1, L87.1
Redo open surgery, long saphenous	L84.4
Primary open surgery, short saphenous	L84.2
Redo open surgery, short saphenous	L84.5
Primary open surgery, both systems	L84.3
Redo open surgery, both systems	L84.6, L85.3
Sclerotherapy	L86.1, L86.2, L86.8, L86.9
Endovenous laser therapy and radiofrequency ablation	L88.1, L88.2, L88.3, L88.8, L88.9
Other open surgery (unspecified), ie phlebectomy	L84.8, L84.9, L85.9, L87.1, L87.3, L87.4, L87.5, L87.6, L87.7, L87.8, L87.9, L85.8

## Results

A overall of 35,374 patients (65% female, median age: 50 years, interquartile range [IQR]: 39–60 years) were identified in this study. Three-quarters (74%) had their procedure performed as a day case. The median length of stay for the remaining patients was 1 day (range: 1–143 days).

The overall incidence of VTE in our study was 0.51%. A total of 126 patients reattended with a post-operative DVT (0.36%) and 53 with a PE (0.15%). Half (51%) of these individuals were female with a median age of 54 years (IQR: 45–62 years). The median time to re-presentation with DVT and PE was 11 days (IQR: 0–77 days) and 18 days (IQR: 8–48 days) respectively.

Table 2 shows the incidence of VTE, which is comparable with those undergoing open surgery (0.54%), sclerotherapy (0.19%) and EVLT (0.47%). The incidence of VTE in those undergoing a combination of EVLT and phlebectomy, however, was 1.26% (chi-squared test, *p*=0.01). The difference in incidence of VTE in this cohort (1.26%) compared directly with those who underwent EVLT alone (0.47%) was also found to be statistically significant (*p*=0.05).

**Table 2:** Treatments performed and incidence of venous thromboembolism

Analysis by	Number of procedures	Number of DVTs	Number of PEs	Total VTE episodes
*Limb*			
Unilateral	28,947 (81.8%)	86 (0.30%)	44 (0.15%)	130 (0.45%)
Bilateral	6,427 (18.2%)	40 (0.62%)	9 (0.14%)	49 (0.76%)
*Attempt*			
Primary	32,674 (92.4%)	113 (0.35%)	48 (0.15%)	161 (0.49%)
Redo	2,700 (7.6%)	13 (0.48%)	5 (0.19%)	18 (0.67%)
*System*			
Long	21,144 (59.8%)	77 (0.36%)	36 (0.17%)	113 (0.53%)
Short	1,493 (4.2%)	9 (0.60%)	1 (0.07%)	10 (0.67%)
Both	1,832 (5.2%)	9 (0.49%)	4 (0.22%)	13 (0.71%)
Unknown	10,905 (30.8%)	31 (0.28%)	12 (0.11%)	43 (0.39%)
*Modality*			
Open	29,435 (83.2%)	108 (0.37%)	50 (0.17%)	158 (0.54%)
EVLT	1,499 (4.2%)	6 (0.40%)	1 (0.07%)	7 (0.47%)
EVLT + phlebectomy	557 (1.6%)	7 (1.26%)	0 (0.00%)	7 (1.26%)
Sclerotherapy	3,701 (10.5%)	5 (0.14%)	2 (0.05%)	7 (0.19%)
Sclerotherapy + phlebectomy	71 (0.2%)	0 (0.00%)	0 (0.00%)	0 (0.00%)
EVLT + sclerotherapy	111 (0.3%)	0 (0.00%)	0 (0.00%)	0 (0.00%)

EVLT = endovenous laser therapy; DVT = deep vein thrombosis; PE = pulmonary embolism; VTE = venous thromboembolism

A total of 360 cases (1.02%) were performed under local anaesthesia with zero incidence of VTE in this cohort. There was no overall significant difference in VTE incidence between those patients having primary or redo surgery, nor between those undergoing long or short saphenous system surgery.

Where unilateral surgery was performed, there was no difference in the incidence of VTE between left and right limbs. Those patients undergoing bilateral surgery had more than double the risk of DVT than those having unilateral surgery (0.62% vs 0.30%). Where bilateral surgery was performed, a statistically significant increase in the incidence of VTE was seen in those undergoing redo surgery of 0.96 percentage points (95% confidence interval: 0.21–2.23%), from 0.66% to 1.62%. Similarly, in those patients undergoing bilateral short saphenous system surgery, an increase in VTE incidence of 0.44 percentage points from 0.72% to 1.16% was observed if the case involved redo surgery. Two deaths were identified in the study; neither were coded as having suffered from VTE.

## Discussion

Varicose vein surgery in its many modalities remains a commonly performed procedure and the incidence of VTE in our large national study was 0.51%. There are a number of limitations for studies using HES data. The two most striking are the potential for miscoding and an underestimation of the total number of VTE episodes due to patients not being admitted to hospital. Nevertheless, the quality of HES data has improved greatly in recent years^7^ and HES has been shown as a useful tool to measure effectiveness and for comparative audit.^8^ It has also been shown in a systematic review that there is a high level of accuracy for diagnosis.^9^

The data suggest that those patients undergoing a combination of EVLT and phlebectomy may be at the greatest risk of VTE (1.26%) although the numbers in this group from the 2006–2007 dataset are relatively small (*n*=557), reflecting the gradual uptake of EVLT into NHS practice. Present restrictions on patient access to NHS varicose vein treatment may make re-examination of this point difficult. Patients having bilateral surgery are at higher risk of VTE, which is most marked in those undergoing either redo (1.62%) or short saphenous system (1.16%) surgery. In addition, all cases of VTE were reported in patients who underwent general anaesthesia. What is not clear from the data available is whether this is related to the length of procedure or other factors known to contribute to VTE such as dehydration, post-operative pain or immobility.^4^

## Conclusions

We have identified subsets of patients who appear to be at greater risk of VTE following varicose vein surgery: those undergoing general anaesthesia and either EVLT combined with phlebectomy or bilateral surgery. There are no universal guidelines on the use of peri-operative thromboprophylaxis for these cohorts and it cannot be determined from HES whether VTE prophylaxis was used for individual cases. Our data support the use of VTE prophylaxis in all patients undergoing general anaesthetic varicose vein surgery, but in particular for these seemingly higher risk groups.
